# Brain structural changes in cynomolgus monkeys administered with 1-methyl-4-phenyl-1,2,3,6-tetrahydropyridine: A longitudinal voxel-based morphometry and diffusion tensor imaging study

**DOI:** 10.1371/journal.pone.0189804

**Published:** 2018-01-10

**Authors:** Hyeonseok S. Jeong, Sang-Rae Lee, Jieun E. Kim, In Kyoon Lyoo, Sujung Yoon, Eun Namgung, Kyu-Tae Chang, Bom Sahn Kim, Sejung Yang, Jooyeon J. Im, Saerom Jeon, Ilhyang Kang, Jiyoung Ma, Yong-An Chung, Soo Mee Lim

**Affiliations:** 1 Department of Radiology, Incheon St. Mary’s Hospital, College of Medicine, The Catholic University of Korea, Seoul, South Korea; 2 National Primate Research Center, Korean Research Institute of Bioscience and Biotechnology, Ochang, South Korea; 3 Ewha Brain Institute, Ewha Womans University, Seoul, South Korea; 4 Department of Brain and Cognitive Sciences, Ewha Womans University, Seoul, South Korea; 5 Graduate School of Pharmaceutical Sciences, Ewha Womans University, Seoul, South Korea; 6 Department of Nuclear Medicine, Ewha Womans University Mokdong Hospital, Seoul, South Korea; 7 Institute of Convergence Medicine, Ewha Womans University Medical Center, Seoul, South Korea; 8 Interdisciplinary Program in Neuroscience, College of Natural Sciences, Seoul National University, Seoul, South Korea; 9 Department of Radiology, Ewha Womans University Mokdong Hospital, Seoul, South Korea; Georgia Regents University, UNITED STATES

## Abstract

In animal models of Parkinson's disease (PD), 1-methyl-4-phenyl-1,2,3,6-tetrahydropyridine (MPTP) is one of the most widely used agents that damages the nigrostriatal dopaminergic pathway. However, brain structural changes in response to MPTP remain unclear. This study aimed to investigate *in vivo* longitudinal changes in gray matter (GM) volume and white matter (WM) microstructure in primate models administered with MPTP. In six cynomolgus monkeys, high-resolution magnetic resonance imaging (MRI) and diffusion tensor imaging (DTI) scans were acquired 7 times over 32 weeks, and assessments of motor symptoms were conducted over 15 months, before and after the MPTP injection. Changes in GM volume and WM microstructure were estimated on a voxel-by-voxel basis. Mixed-effects regression models were used to examine the trajectories of these structural changes. GM volume initially increased after the MPTP injection and gradually decreased in the striatum, midbrain, and other dopaminergic areas. The cerebellar volume temporarily decreased and returned to its baseline level. The rate of midbrain volume increase was positively correlated with the increase rate of motor symptom severity (Spearman rho = 0.93, p = 0.008). Mean, axial, and radial diffusivity in the striatum and frontal areas demonstrated initial increases and subsequent decreases. The current multi-modal imaging study of MPTP-administered monkeys revealed widespread and dynamic structural changes not only in the nigrostriatal pathway but also in other cortical, subcortical, and cerebellar areas. Our findings may suggest the need to further investigate the roles of inflammatory reactions and glial activation as potential underlying mechanisms of these structural changes.

## Introduction

Parkinson's disease (PD) is the second most common neurodegenerative disorder and is primarily characterized by progressive loss of dopaminergic neurons in the substantia nigra (SN), decreased levels of striatal dopamine, and consequent development of motor symptoms [1].

The neurotoxin 1-methyl-4-phenyl-1,2,3,6-tetrahydropyridine (MPTP) has been most widely used as a potent agent to investigate the pathophysiology of PD in animal models. However, *in vivo* brain structural changes in response to MPTP remain unclear in spite of a growing number of functional and molecular imaging studies. Voxel-based morphometry (VBM) analysis of structural magnetic resonance imaging (MRI) data allows reliable quantification of regional gray matter (GM) volumes in an unbiased way [2]. In addition, diffusion tensor imaging (DTI) is a promising imaging modality for mapping three-dimensional diffusion of water in biologic tissues and characterizing microstructural changes in white matter (WM). Therefore, longitudinal studies using both VBM and DTI methods may provide new insight into MPTP-induced brain structural changes and the pathophysiology of PD.

Two recent studies in MPTP-treated primate models provided histopathological evidence for the validity of VBM and DTI techniques and demonstrated changes in GM volume and WM microstructure [3, 4]. However, the follow-up MRI scans were performed only once at 10 weeks after MPTP injection in these studies [3, 4]. The current multimodal imaging study aimed to investigate long-term trajectories of GM volume and WM microstructure and their associations with motor symptoms in MPTP-administered monkeys using high-resolution MRI and DTI scans and standardized assessment of PD motor symptoms.

Increasing evidence has suggested that inflammatory responses and glial activation play pivotal roles in both the mechanism of MPTP toxicity and etiology of PD [5]. Previous MRI studies reported T2 hyperintensities in the SN and striatum in MPTP-administered animals, suggesting selective cytotoxic edema in these regions [6, 7]. Furthermore, a series of histopathological studies have consistently reported that MPTP induces robust and transient gliosis, followed by degeneration of dopaminergic neurons in the SN and striatum [8–11]. Proliferation and/or hypertrophy of glial cells are generally considered possible reasons for GM volume increase in VBM research [12, 13]. Therefore, we hypothesized that GM volume in the nigrostriatal areas would temporarily increase soon after MPTP administration and subsequently decrease.

Regarding the potential associations between the GM volume changes and motor symptom severity, a previous positron emission tomography (PET) study suggested that microglial activity in the midbrain is positively correlated with the symptom severity in patients with early PD [14]. In previous preclinical studies, each MPTP-administered monkey showed a distinctive disease progression pattern despite the administration of the same dosage [15]. Specifically, initial increase in motor symptom severity and spontaneous partial recovery were found with different time course and magnitude. Therefore, we expected that the rates of change in the brain volumes and motor symptoms rather than the differences themselves may provide better explanations for the relationships between them. Our second hypothesis was that faster volume increase in the nigrostriatal regions would be associated with an increased rate of change in motor symptom severity during the early phase.

A previous DTI study in primate models found non-significant changes in fractional anisotropy (FA) value and increased mean diffusivity (MD), axial diffusivity (AD), and radial diffusivity (RD) values in the nigrostriatal pathways at 10 weeks after MPTP injection [4]. FA is a measure of directionality of diffusion and reflects fiber microstructural integrity. MD is an average of the eigenvalues of the diffusion tensor and related to altered membrane density or tissue degeneration. The nature of diffusion parallel or perpendicular to WM tracts are referred to as AD and RD, respectively. AD is specific to axonal injury while RD is sensitive to demyelination. Detailed definitions of these measures and their relationships to WM pathologic features are summarized in a previous review [16]. Considering partial recovery of clinical symptoms in later stages of MPTP-induced PD [15], we examined whether the increases in MD, AD, and RD values of the nigrostriatal WM tracts are alleviated in the long term.

## Materials and methods

### Experimental animals and MPTP administration

Six female cynomolgus monkeys (*Macaca fascicularis*) were used in the study. Their mean age and mean body weight at the baseline MRI scans were 5.6 ± 1.0 years and 3.3 ± 0.3 kg, respectively. The monkeys originated from the Zhaoqing Laboratory Animal Research Centre (Guangdong Province, China) and were maintained in individual indoor cages at the National Primate Research Center in Korea Research Institute of Bioscience and Biotechnology (KRIBB) as previously reported [17, 18]. Food enrichment was provided. Water, monkey chow (Harlan, USA), and various fresh fruits including apples, bananas, and grapes were supplied *ad libitum*. Environmental conditions were controlled with a temperature of 24 ± 2°C, a relative humidity of 50 ± 5%, and a 12:12 h light:dark cycle. The health of the monkeys was monitored by the attending veterinarian in accordance with the recommendations of the Weatherall Report. They were also monitored by microbiological tests including B virus, simian retrovirus (SRV), simian immunodeficiency virus (SIV), simian virus 40 (SV40), and simian T-cell lymphotropic virus (STLV) every year. After the baseline MRI scans, MPTP-HCl (Sigma-Aldrich, St. Louis, MO, USA) was administered by intramuscular injections once a week over the course of five weeks, as daily injections of MPTP can be fatal. The dosage of MPTP-HCl for each administration was 0.45 mg/kg, and thus, the cumulative dose was 2.25 mg/kg [19, 20].

### Ethics statement

All procedures and animal use were approved by the KRIBB Institutional Animal Care and Use Committee (Approval No. KRIBB-AEC-16068). Extensive efforts were made to minimize animal suffering and to reduce the number of animals used.

### Parkinsonian symptom assessment

The Parkinsonian motor symptoms were evaluated according to the rating scale for monkey PD models [21]. This scale evaluates nine items: alertness, head checking movement, eye blinking and movement, posture, balance, motility at rest, reactive motility to external stimuli, walking, and tremor. A higher total score indicates more severe motor impairment. The assessment was performed by a well-trained examiner who was blind to the purpose of the study, at baseline and 1, 3, 6, 9, and 15 months after the first administration of MPTP. An additional 11 to 12 evaluations with different intervals were performed during the first 3 months with a mean interval of 8.6 (standard deviation, SD = 7.2) days.

### Brain magnetic resonance imaging

As described in our previous report [18], MRI scans were acquired at baseline and 8, 16, 20, 24, 28, and 32 weeks after the first administration of MPTP. The monkeys were initially anesthetized with intramuscular injections of 0.5 mg/kg ketamine (Yuhan, Seoul, South Korea). During MRI scanning, monkeys were anesthetized with 2% isoflurane (Hana Pharmacy, Hwaseong, South Korea) in 99.9% oxygen (2 L/min) using an anesthesia machine (Royal Medical, Gyeonggi, South Korea) and immobilized in a supine position in a custom-made bed holder. Inhaled CO_2_ level, O_2_ saturation, pulse, respiration rate, and body temperature were monitored continuously, and body temperature was maintained with a warm blanket surrounding the animal. All MRI experiments were performed on a Philips 3T Achieva scanner (Philips Medical System, Best, Netherlands) with a 32-channel head coil. Three-dimensional sagittal T1-weighted images with a voxel size of 0.25 x 0.25 x 0.50 mm and DTI data were acquired. Detailed acquisition protocols are presented in S1 Text of the Supporting Information. Although no animals were sacrificed for the study, one died from anesthetic complications after the MRI scan at week 32. Due to technical problems with storage at week 16, two T1-weighted images and three DTI scans were lost. In addition, one DTI scan at baseline was excluded from the analysis due to severe artifacts.

### Image processing

We used an iterative process to create a study-specific T1-weighted template and its associated tissue probability maps from the baseline T1-weighted images, based on previous MRI studies in primate models [22, 23]. Detailed procedures are demonstrated in S1 Text of the Supporting Information.

Voxel-based morphometry analysis was performed in Statistical Parametric Mapping version 8 (SPM; Wellcome Department of Cognitive Neurology, Institute of Neurology, London, UK). For each subject, all follow-up scans were realigned to the baseline scan. The mean reference image for each subject was calculated from the baseline images and realigned follow-up images from the previous step. Intra-subject bias correction was conducted for each time point with regard to the reference image. All reference images and bias-corrected images were segmented into GM, WM, and cerebrospinal fluid (CSF). The spatial normalization parameters of the diffeomorphic anatomical registration through exponentiated lie algebra (DARTEL) registration algorithm [24] were estimated from the GM and WM segments from the mean reference images and applied to the GM tissue segments from the bias-corrected baseline and follow-up images. The resulting GM segments were smoothed with a 2 mm full-width half-maximum (FWHM) Gaussian kernel.

We processed and analyzed DTI images as suggested by the previous DTI study in MPTP-administered monkeys [4]. Individual FA, MD, AD, and RD maps were acquired after the skull-stripping and correction of eddy current distortions using FMRIB's Diffusion Toolbox in FSL (http://www.fmrib.ox.ac.uk/fsl). All these maps were spatially normalized to our T1-weighted template and smoothed with a 2 mm FWHM Gaussian kernel.

Visual assessments were conducted at each processing step to control the quality of the processed images.

### Statistical analysis

Changes in motor symptom score from baseline to 15 months were assessed by the Friedman test with post-hoc pairwise Wilcoxon signed rank test. For each subject, we selected the maximum scores between baseline and week 8 as the representative scores for week 8, while the maximum scores between week 8 and week 16 were chosen as the summary scores for week 16 for the following reasons: (1) Different numbers of symptom assessments were performed with different intervals for each animal from baseline to 3 months; (2) The MPTP injections were performed over the first five weeks; (3) Spontaneous partial recovery of motor symptoms are common in MPTP-injected animals [15].

Changes in GM volume from week 8 through week 32 were analyzed on a voxel-by-voxel basis using within-subject one-way ANOVA. The height threshold was set at p < 0.05 for each voxel. Cluster size was thresholded at family-wise error (FWE) corrected p < 0.05 based on Gaussian random field theory [25]. For each significant cluster at week 8, GM volume was extracted at all time points using MarsBar toolbox version 0.44 (http://marsbar.sourceforge.net). Normalized GM volume at each time point was calculated by dividing the regional GM volume by total GM volume and then multiplying it by 100.

Changes in four DTI measures (FA, MD, AD, and RD) from week 8 through week 32 were also examined using within-subject one-way ANOVA. The statistical threshold was p < 0.05 at voxel level and a minimum of 150 voxels. For each measure, MarsBar toolbox was used to extract the mean values in the significant clusters at week 8 over all time points.

In order to determine entire trajectories of normalized GM volumes or DTI measures at the significant clusters, mixed model regression analyses were conducted over 32 weeks, using linear and quadratic models. The Akaike information criterion (AIC)[26] and Bayesian information criterion (BIC)[27] were used to assess overall model fit and to select the best-fitting model. Statistics were estimated from 1,000 bootstrapped samples in order to compensate for the small sample size distortion [28].

For correlation analysis between the GM volume and symptom severity, the rate of change for each measure was defined as the maximum change divided by the corresponding time interval in weeks. The associations between the rate of the GM volume change and that of the symptom severity increase were evaluated with Spearman correlation coefficients. In the secondary correlation analysis, the average scores between week 5 and week 8 and between week 8 and week 16 were selected as the summary scores for week 8 and week 16 for each animal, respectively.

Statistical tests were conducted using the STATA version 13.1 (StataCorp., College Station, TX, USA) and were two-tailed with an alpha level of 0.05.

## Results

### Changes in motor symptom severity

The scores of the motor symptom severity were 0.0 (SD = 0.0) at the baseline, 3.5 (SD = 3.4) at week 8, 4.2 (SD = 3.9) at week 16, 2.5 (SD = 2.4) at 6 months, 2.6 (SD = 2.2) at 9 months, and 2.6 (SD = 2.2) at 15 months (S1 Fig). The change of the symptom score was significant in the Friedman test (p = 0.004). In post-hoc tests, only the change between baseline and week 8 was significant (z = 2.21, p = 0.027), although the mean score increased up to 16 weeks and remained stable from 9 months onwards after the partial recovery.

### Changes in gray matter volume

Results of the VBM analysis identified three clusters with significant GM volume changes at week 8 compared with baseline (Table 1 and S2 Fig). The first cluster, which included the striatum (caudate, putamen, and nucleus accumbens) and surrounding areas such as the amygdala, hypothalamus, and frontal and lateral orbital gyrus, bilaterally, demonstrated the GM volume increase (t = 11.1, p < 0.001, cluster size = 400 mm^3^). The second cluster that revealed the GM volume increase (t = 9.5, p < 0.001, cluster size = 359 mm^3^) encompassed the midbrain and nearby regions including the cuneus, fusiform gyrus, and parahippocampal gyrus, bilaterally. The third cluster indicated reduced GM volume in the cerebellum (t = 9.0, p < 0.001, cluster size = 707 mm^3^).

**Table 1:** Clusters with significant gray matter volume changes at week 8.

Cluster	Region	t	p (voxel level)	Cluster size (mm^3^)	p (cluster level)^a^
*Baseline < week 8*					
1	Bilateral striatum, amygdala, hypothalamus, frontal and lateral orbital gyrus	11.1	< 0.001	400	0.007
2	Bilateral midbrain, cuneus, fusiform gyrus, and parahippocampal gyrus	9.5	< 0.001	359	0.015
*Baseline > week 8*					
3	Bilateral cerebellum	9.0	< 0.001	707	< 0.001

^a^ Family-wise error corrected p-value.

**Fig 1:**
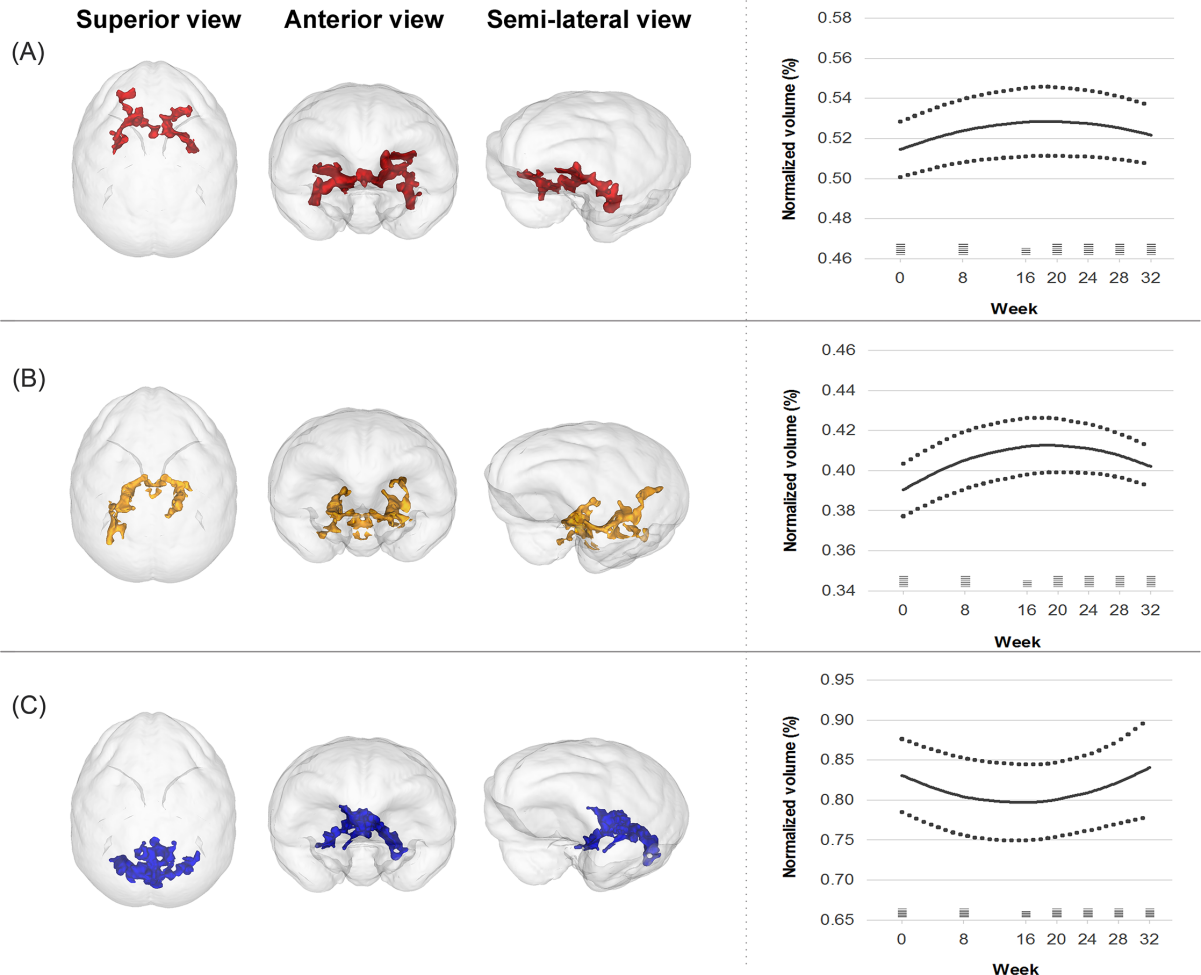
**Trajectories of gray matter volume changes in the first (A), second (B), and third (C) clusters over 32 weeks.** Solid lines represent the fitted regression lines and dotted lines show the 95% confidence intervals for the regression lines. Horizontal bars at each time point demonstrate the sample size.

In the regression analysis over the 32 weeks, the quadratic models provided a better fit than the linear models for the volume trajectories of the first cluster (linear model: Wald χ^2^ = 11.6, p = 0.001, AIC = -224.6, BIC = -217.8; quadratic model: Wald χ^2^ = 11.7, p = 0.003, AIC = -228.9, BIC = -220.5) and the second cluster (linear model: Wald χ^2^ = 9.2, p = 0.003, AIC = -220.7, BIC = -213.9; quadratic model: Wald χ^2^ = 92.1, p < 0.001, AIC = -233.2, BIC = -224.8). For the third cluster, only the quadratic model was significant (linear model: Wald χ^2^ = 0.1, p = 0.79; quadratic model: Wald χ^2^ = 20.9, p < 0.001). The GM volumes in the first and second clusters increased up to approximately 18 weeks after MPTP injection and gradually decreased, whereas the volume of the third cluster decreased up to approximately 15 weeks and increased afterwards (Fig 1).

In the primary correlation analysis, the increased rate of the GM volume in the second cluster was positively correlated with the increased rate of the motor symptom severity (Spearman rho = 0.93, p = 0.008). However, the first (p = 0.827) and third clusters (p = 0.257) did not show such relationships. In the secondary correlation analysis, correlation was still significant between the rate of the GM volume increase in the second cluster and that of the symptom severity increase (Spearman rho = 0.84, p = 0.036). The first (p = 0.957) and third clusters (p = 0.173) were not related to the symptom score.

### Changes in white matter microstructure

**Fig 2:**
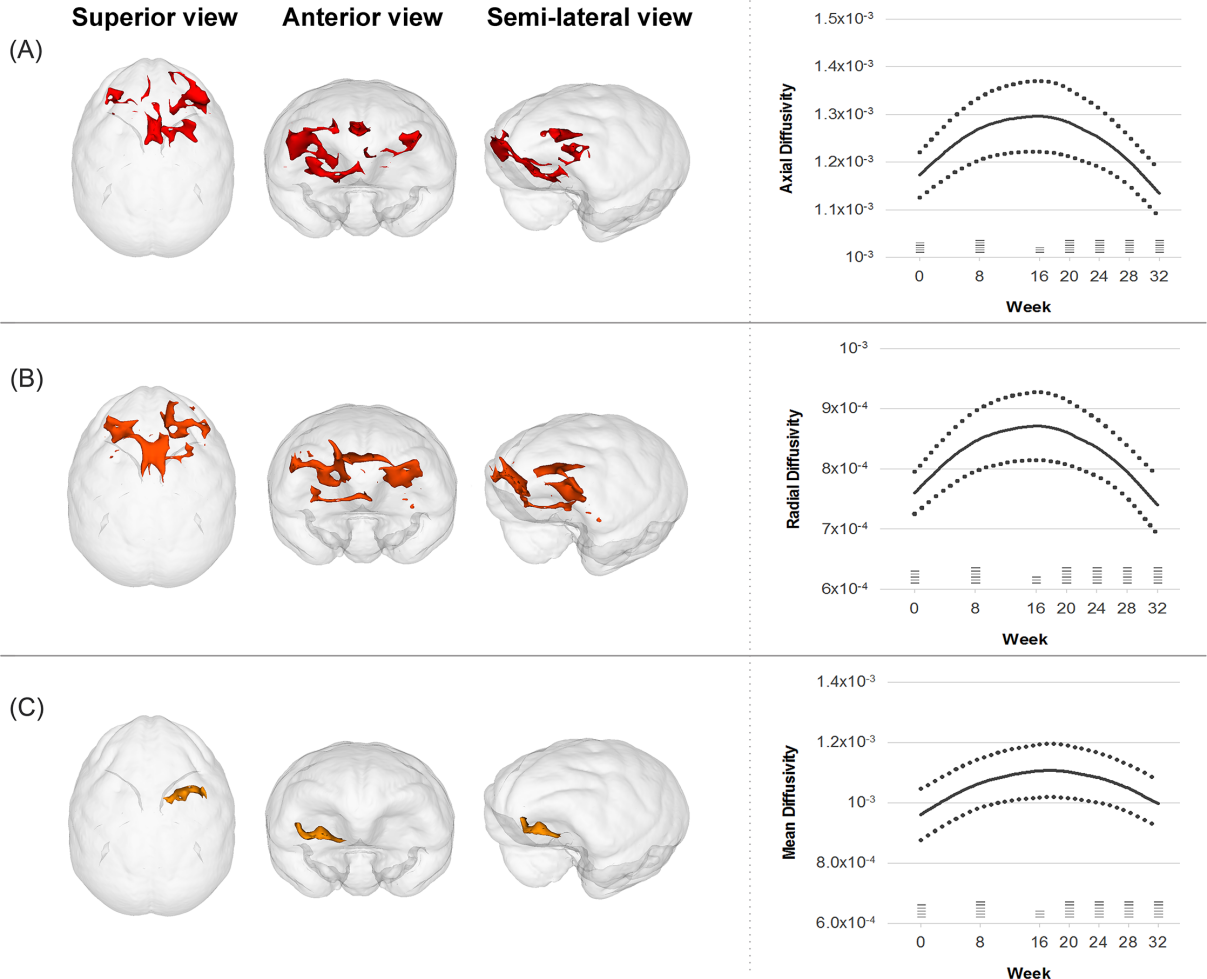
**Trajectories of changes in diffusion tensor imaging parameters including axial diffusivity (A), radial diffusivity (B), and mean diffusivity (C) over 32 weeks.** Solid lines represent the fitted regression lines and dotted lines show the 95% confidence intervals for the regression lines. Horizontal bars at each time point demonstrate the sample size.

FA values did not significantly change in any regions at week 8 (S3 Fig). The AD value increased in four clusters located in the bilateral striatum and orbito/inferior frontal regions at week 8 (t = 6.9, p = 0.001, total cluster size = 864 mm^3^)(S4 Fig). The mean AD value of these clusters showed a temporary increase followed by a decrease over 32 weeks and was fitted to a quadratic curve (linear model: Wald χ2 = 20.0, p < 0.001, AIC = -577.6, BIC = -571.0; quadratic model: Wald χ2 = 22.4, p < 0.001, AIC = -589.4, BIC = -581.2)(Fig 2A).

The RD value increased in two clusters located in the bilateral striatum and orbito/inferior frontal areas (t = 7.8, p = 0.001, total cluster size = 892 mm^3^) and decreased in three clusters located in the right temporal and bilateral superior frontal WM (t = 6.3, p = 0.002, total cluster size = 508 mm^3^) at week 8 (S5 Fig). The mean RD of the increased clusters transiently increased and then decreased over 32 weeks, showing a quadratic curve (linear model: Wald χ2 = 3.3, p = 0.071; quadratic model: Wald χ2 = 30.2, p < 0.001)(Fig 2B). However, the mean RD of the decreased cluster was not fitted to neither the linear (Wald χ2 = 0.4, p = 0.523) nor quadratic models (Wald χ2 = 2.8, p = 0.248).

The WM tracts near the right striatum showed a significant increase in MD value (t = 8.2, p = 0.001, cluster size = 158 mm^3^)(S6 Fig). The MD value increased up to 16 weeks and subsequently decreased by 32 weeks, showing a pattern fitted to a quadratic curve (linear model: Wald χ2 = 0.8, p = 0.370; quadratic model: Wald χ2 = 17.0, p < 0.001)(Fig 2C).

## Discussion

In this longitudinal *in vivo* multi-modal neuroimaging study of cynomolgus monkeys, we investigated MPTP-induced changes in brain structures and their associations with motor symptoms. The major strengths of the current study are the large number of high-resolution MRI and DTI images and standardized behavioral assessments over a long period. Our main findings revealed that MPTP injection induced a transitory volume increase in the midbrain, striatum, and other major dopaminergic areas, and a temporary volume reduction in the cerebellum. The rate of the midbrain volume increase was positively correlated with the increase rate of motor symptom severity. In addition, initial increases and later decreases in measures of MD, AD, and RD were found in the striatum and frontal areas. Consistent with our results, deficits in the frontal, striatal, midbrain, and cerebellar regions are known to be implicated in the pathophysiology of PD [29, 30].

The GM volumes of the two clusters, which are mainly located in the midbrain and striatum, initially increased after MPTP injection, then gradually decreased afterwards. The nigrostriatal dopaminergic pathway plays a central role not only in the pathophysiology of PD, but also in MPTP neurotoxicity [31]. Specifically, the midbrain and striatal dopaminergic function is impaired in human PD patients [29]. Previous literature has found robust and transient inflammatory reactions and gliosis in the nigrostriatal areas of MPTP-induced PD preclinical models [8, 9]. In addition, glial reactions can contribute to GM volume increase [12, 13]. For instance, increased levels of CSF sTREM2, a marker of microglial activation, are associated with GM volume increases in patients with early Alzheimer's disease [32]. Therefore, we speculate that the volume changes in the midbrain and striatum may be primarily driven by glial activation in the initial phase and subsequent neurodegeneration, although the underlying microscopic mechanism should be further clarified. Measuring CSF levels of inflammatory cytokine or *in vivo* PET imaging of neuroinflammation [33] combined with MRI, may be useful for future studies. Volume changes were not observed in other deep GM structures such as the locus coeruleus, which is also vulnerable to MPTP neurotoxicity [34]. This result may be partly because VBM method can be less sensitive to volumes of small deep brain structures that have low tissue contrast to WM on T1-weighted images [35].

As expected in the second hypothesis, the rate of GM volume increase in the midbrain cluster was highly correlated with the increased rate in the motor symptom score. With regard to the temporal relationship, it is noteworthy that both the volume and score reached their maximum values at a similar time (16 to 18 weeks from baseline). However, the first cluster, which included the striatum, did not show a significant association with the symptom severity. In a previous PET study, the midbrain [^11^C](R)-PK11195 binding potential, a marker for activated microglia, was positively related to motor symptom severity in patients with PD, whereas the striatum did not demonstrate such a relationship [14]. Since the midbrain is the cell body side of the nigrostriatal pathway and the primary target site of MPTP, this region may be more closely associated with the development of MPTP-induced motor symptoms than the striatal area.

The two clusters with increased volume included some parts of other mesolimbic (nucleus accumbens), mesocortical (prefrontal cortex), tuberoinfundibular (hypothalamus), and minor dopaminergic pathways (amygdala and parahippocampal gyrus). MPTP has been known to affect not only the nigrostriatal pathway but also, to a lesser extent, the mesolimbic and mesocortical pathways [36]. In addition, a previous autoradiographic study reported MPTP-induced neurotoxicity in the frontal cortex, amygdala, hippocampus, and hypothalamus in a primate brain [37]. Although the motor symptoms and the nigrostriatal circuits have been the primary interest of PD research, impairment in other dopaminergic systems may also play important roles in the pathophysiology of non-motor PD symptoms such as depression, apathy, and sleep disorders in human patients [38] and primate MPTP models [39]. For instance, dopaminergic alterations in the mesolimbic pathway and the insula are associated with apathy and loss of social behaviors in MPTP-administered monkeys, respectively [40, 41].

The GM volume in the cerebellum decreased in the early phase and returned to its baseline level by 32 weeks. The cerebellum is reciprocally connected to the basal ganglia and increasing evidence demonstrated that the cerebellum may play a role in PD [30]. Consistent with our results, MPTP administration led to loss of cerebellar Purkinje cells in monkeys [42]. The degree of direct dopaminergic innervation in the primate cerebellum, however, is quite restricted [43]. The cerebellar volume decrease in our study may indicate neurodegeneration that is secondary to the MPTP-induced damage in the nigral regions. On the other hand, the subsequent volume increase in the cerebellum is another interesting finding. Although speculative, this increase may be related to the spontaneous partial recovery in motor deficits following MPTP treatment [15, 44]. Previous neuropathological studies in animal models suggested some degree of regeneration of dopaminergic neurons [45] and a compensatory increase in dopamine turnover by surviving neurons after MPTP administration [46].

The MD, AD, and RD values increased up to 16 weeks and decreased afterwards in the fronto-striatal areas that also showed GM volume changes. The increase in AD value may be explained by axonal deterioration. In response to expanded extra-axonal space and lower axonal density, water movements may potentially be faster parallel to the axon [47, 48]. On the other hand, higher RD value may suggest demyelination, neuroinflammation with macrophage infiltration, or edema [49]. Taken together, concurrent increases in AD and RD values may indicate axonal injury in combination with myelin loss. In addition, increased MD is related to higher level of tissue water caused by edema or inflammation. Since FA reflects the degree of anisotropy of a diffusion process, increases in both AD and RD values may reduce the sensitivity of FA. The trajectories of MD, AD, and RD were fitted to quadratic curves as seen in the volumetric changes. Given that trajectories of changes in brain GM and WM structures share similar time frames, we speculate that the normalization processes in both GM volumes and DTI measures may lead to the partial recovery in the clinical symptoms.

Some potential limitations of the study should be addressed. First, although behavioral motor symptoms were induced by MPTP administration as noted in the results, the exact dopaminergic effects caused by MPTP could not be confirmed in the current study. Future studies using *in vivo* dopamine PET imaging would be necessary. Second, although substantial evidence from previous studies supports that strong gliosis may cause the acute volume increase in the nigrostriatal regions after MPTP treatment, the cellular level changes could not be detected by MRI and DTI studies. Thus, a direct causal relationship between them cannot be determined by the results of the current study. Third, it is noted that there was no control subjects and the sample size was relatively small. Therefore, potential confounding factors including the experimental environment and aging effects should be considered in interpreting the current findings. Further larger prospective studies with control animals are warranted. Fourth, the different time points between the brain imaging and clinical evaluation and the loss of MRI and DTI images at week 16 due to technical problems were also limitations.

In summary, the current longitudinal VBM-DTI study of MPTP-administered monkeys revealed widespread and dynamic structural changes not only in the nigrostriatal pathway but also in the other cortical, subcortical, and cerebellar areas. Moreover, the rate of nigral volume increase was associated with the rate of motor symptom development. Our findings may suggest the need to further investigate the roles of inflammatory reactions and glial activation as potential underlying mechanisms of these structural changes. In addition, an interaction between the brain angiotensin system and MPTP would be a valuable topic for future *in vivo* imaging studies, as this system modulates striatal dopamine release, and inflammatory responses by MPTP can be reduced by angiotensin receptor antagonists [50].

## Supporting information

S1 Text Supplementary materials and methods. (DOCX)

S1 Table The ARRIVE guidelines checklist. (PDF)

S1 Fig Changes in motor symptom scores. Black dashed lines represent score of each subject and blue solid line demonstrates the mean score.
(DOCX)

S2 Fig Changes in regional gray matter volume from week 8 to week 32 compared to baseline. Areas with volume increase and decrease appear in red and blue, respectively. Figures at week 16 were omitted due to missing data for 2 subjects. The height threshold was p < 0.05 and extent threshold was family-wise error corrected p < 0.05. All images are shown in neurological convention.
(DOCX)

S3 Fig Changes in fractional anisotropy (FA) from week 8 to week 32 compared to baseline. Areas with FA increase and decrease appear in red and blue, respectively. Figures at week 16 were omitted due to missing data for 3 subjects. The height threshold was p < 0.05 and extent threshold was 150 voxels. All images are shown in neurological convention.
(DOCX)

S4 Fig Changes in axial diffusivity (AD) from week 8 to week 32 compared to baseline. Areas with AD increase and decrease appear in red and blue, respectively. Figures at week 16 were omitted due to missing data for 3 subjects. The height threshold was p < 0.05 and extent threshold was 150 voxels. All images are shown in neurological convention.
(DOCX)

S5 Fig Changes in radial diffusivity (RD) from week 8 to week 32 compared to baseline. Areas with RD increase and decrease appear in red and blue, respectively. Figures at week 16 were omitted due to missing data for 3 subjects. The height threshold was p < 0.05 and extent threshold was 150 voxels. All images are shown in neurological convention.
(DOCX)

S6 Fig Changes in mean diffusivity (MD) from week 8 to week 32 compared to baseline. Areas with MD increase and decrease appear in red and blue, respectively. Figures at week 16 were omitted due to missing data for 3 subjects. The height threshold was p < 0.05 and extent threshold was 150 voxels. All images are shown in neurological convention.
(DOCX)
