# Supplementary material for: Brain structural changes in cynomolgus monkeys administered with 1-methyl-4-phenyl-1,2,3,6-tetrahydropyridine: A longitudinal voxel-based morphometry and diffusion tensor imaging study
Source: PLoS One. 2018 Jan 10;13(1):e0189804. doi: 10.1371/journal.pone.0189804 (PMC5761839; doi:10.1371/journal.pone.0189804)
Supplement: S1 Text — (DOCX) [file pone.0189804.s001.docx]

**Supplementary materials and methods**

**Brain magnetic resonance imaging**

Parameters for T1-weighted scans are as follows: echo time = 6.9 ms, repetition time = 13.9 ms, flip angle = 8°, number of signal averages = 4, acquisition matrix = 260 x 161 mm, field of view = 130 x 130 x 75 mm, voxel size = 0.25 x 0.25 x 0.50 mm. Diffusion tensor imaging (DTI) scans were also performed using a spin-echo, echo-planar diffusion-weighted sequence (echo time = 48 ms, repetition time = 5700 ms, flip angle = 90°, field of view = 96 x 96 mm, acquisition matrix = 64 x 63 mm, voxel size = 1.5 x 1.5 x 1.5 mm, b = 700 s/mm^2^, 128 directions).

**Creation of T1-weighted template and tissue priors**

All images were resliced to a voxel size of 0.25 x 0.25 x 0.25 mm and skull-stripped using Brain Extraction Tool [1] in FMRIB Software Library (FSL) version 5.0.9 (www.fmrib.ox.ac.uk/fsl). Additional manual editing was conducted for accurate brain extractions. After five baseline images were registered to a single representative baseline scan using Statistical Parametric Mapping version 8 (SPM; Wellcome Department of Cognitive Neurology, Institute of Neurology, London, UK), all baseline images were averaged to create an initial template. Then, all baseline scans were segmented into gray matter (GM), white matter (WM), and cerebrospinal fluid (CSF) using FAST [2], which does not need tissue priors. All baseline T1-weighted volumes were spatially normalized to the initial template using SPM and resulting normalization parameters for each monkey were applied to the segmented GM, WM, and CSF images. The normalized T1-weighted images were averaged to create the final version of the T1-weighted template. In addition, the GM, WM, and CSF segments were binarized and averaged to create the tissue probability maps for each tissue type. Finally, three types of tissue priors were smoothed with a 1 mm full-width half-maximum (FWHM) isotropic Gaussian kernel, considering the small voxel size [3]. Visual examinations were performed at each step to control the quality of the processed images.

**References for the supporting information**

1. Smith SM. Fast robust automated brain extraction. Hum Brain Mapp. 2002;17(3):143-55. Epub 2002/10/23. doi: 10.1002/hbm.10062. PubMed PMID: 12391568.

2. Zhang Y, Brady M, Smith S. Segmentation of brain MR images through a hidden Markov random field model and the expectation-maximization algorithm. IEEE Trans Med Imaging. 2001;20(1):45-57. Epub 2001/04/11. doi: 10.1109/42.906424. PubMed PMID: 11293691.

3. McLaren DG, Kosmatka KJ, Oakes TR, Kroenke CD, Kohama SG, Matochik JA, et al. A population-average MRI-based atlas collection of the rhesus macaque. Neuroimage. 2009;45(1):52-9. Epub 2008/12/09. doi: 10.1016/j.neuroimage.2008.10.058. PubMed PMID: 19059346; PubMed Central PMCID: PMCPMC2659879.
