# Supplementary material for: Brain structural changes in cynomolgus monkeys administered with 1-methyl-4-phenyl-1,2,3,6-tetrahydropyridine: A longitudinal voxel-based morphometry and diffusion tensor imaging study
Source: PLoS One. 2018 Jan 10;13(1):e0189804. doi: 10.1371/journal.pone.0189804 (PMC5761839; doi:10.1371/journal.pone.0189804)
Supplement: S1 Fig — Black dashed lines represent score of each subject and blue solid line demonstrates the mean score. (DOCX) [file pone.0189804.s003.docx]

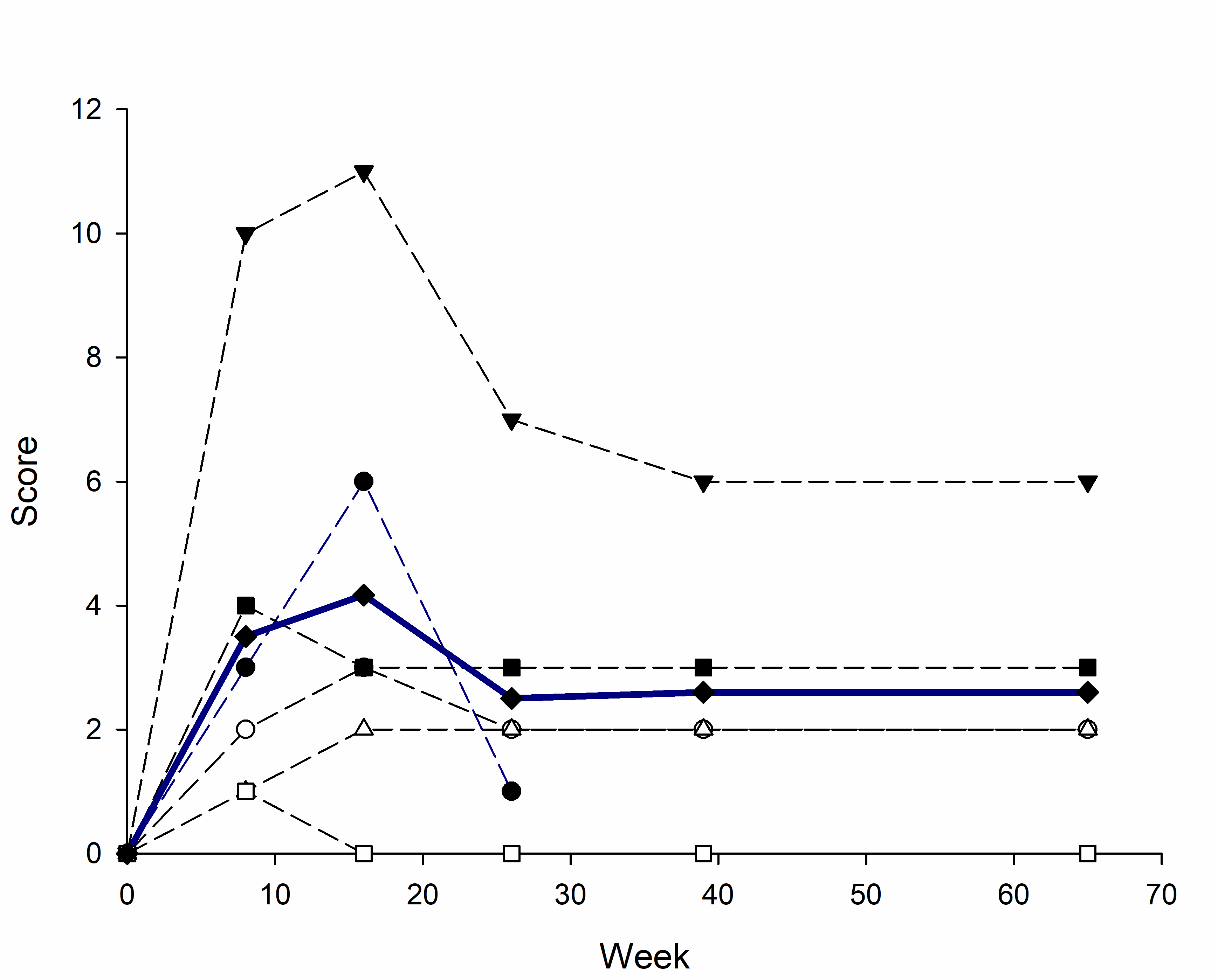


**S1 Fig. Changes in motor symptom scores.** Black dashed lines represent score of each subject and blue solid line demonstrates the mean score.
