# Supplementary material for: Brain structural changes in cynomolgus monkeys administered with 1-methyl-4-phenyl-1,2,3,6-tetrahydropyridine: A longitudinal voxel-based morphometry and diffusion tensor imaging study
Source: PLoS One. 2018 Jan 10;13(1):e0189804. doi: 10.1371/journal.pone.0189804 (PMC5761839; doi:10.1371/journal.pone.0189804)
Supplement: S4 Fig — Areas with AD increase and decrease appear in red and blue, respectively. Figures at week 16 were omitted due to missing data for 3 subjects. The height threshold was p < 0.05 and extent threshold was 150 voxels. All images are shown in neurological convention. (DOCX) [file pone.0189804.s006.docx]

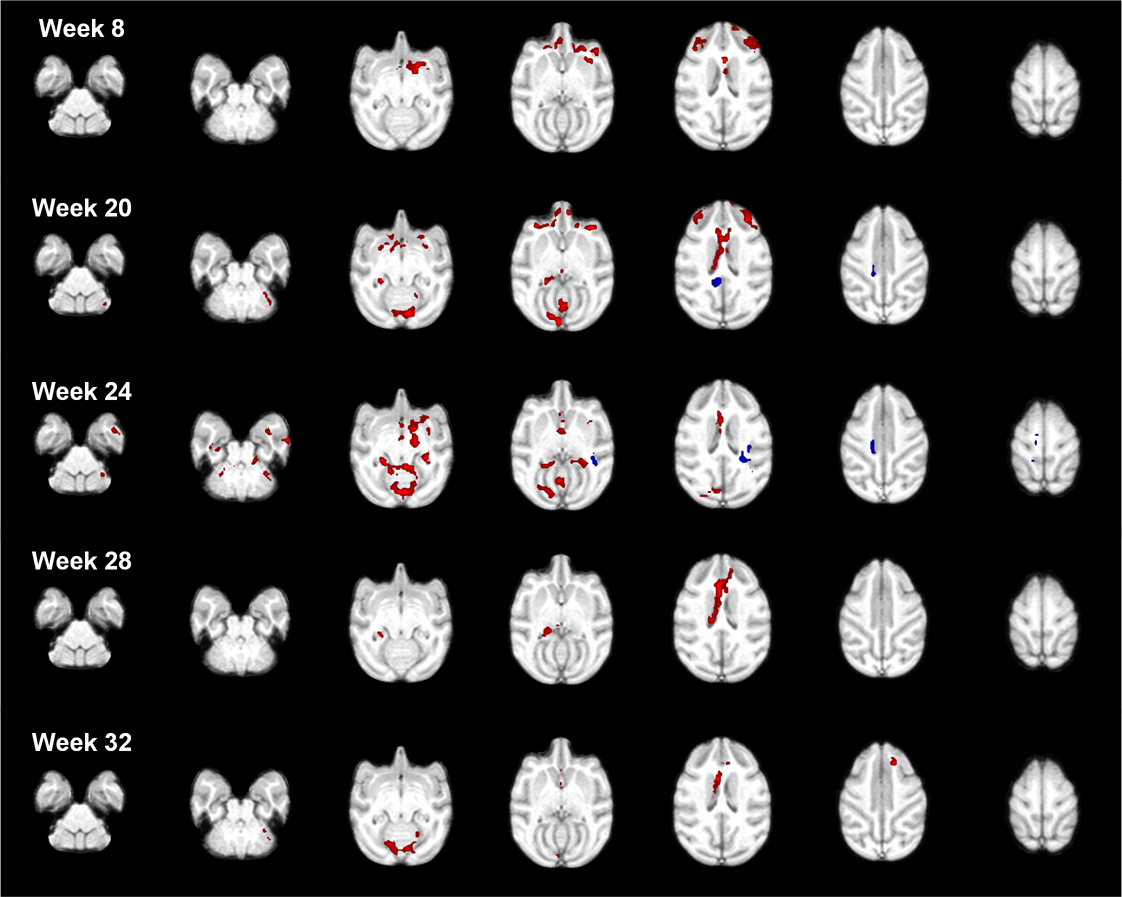


**S4 Fig.** **Changes in axial diffusivity (AD) from week 8 to week 32 compared to baseline.** Areas with AD increase and decrease appear in red and blue, respectively. Figures at week 16 were omitted due to missing data for 3 subjects. The height threshold was p < 0.05 and extent threshold was 150 voxels. All images are shown in neurological convention.
